# Supplementary material for: Real-Life Characteristics of Patients with COPD and Discordant CAT-mMRC Questionnaires
Source: J Clin Med. 2025 Dec 11;14(24):8771. doi: 10.3390/jcm14248771 (PMC12733573; doi:10.3390/jcm14248771)
Supplement: Supplementary file 1 [file jcm-14-08771-s001.zip › master.pdf]

Supplementary Materials: Real-Life Characteristics of Patients with COPD and Discordant CAT-mMRC Questionnaires

Andrea Portacci [1,†], Vitaliano Nicola Quaranta [1,†], Alessio Marinelli [1], Alessandro Capuano [1], Maria Rosaria Vulpi [1], Fabrizio Diaferia [1], Carla Santomasi [1], Maria Francesca Grimaldi [1], Giovanni Sanasi [1], Marianna Cicchetti [1], Eustachio Ricciardi [1], Giulia Amoruso [1], Alfredo Vozza [2], Silvano Dragonieri [1,*] and Giovanna Elisiana Carpagnano [1]

Table S1: Clinical and functional features of the overall cohort according to GOLD classes.

	GOLD A	GOLD B	GOLD E	GOLD D	P value
**Patients (%, n)**	24.8 (55)	34.2 (76)	28.4 (63)	12.6 (28)	
**Age (Years, Median, IQR)**	68 [61-74]	72 [63.2-79]	73 [64-78]	69.5 [63.7-77.2]	0.2
**Gender (Male/Female, %)**	78.2/21.8	80.3/19.7	73/27	92.9/7.1	0.19
**BMI (Days, Median, IQR)**	26 [23-29]	29 [25-33.7]	28 [24.9-33]	25 [22.5-27.3]	**0.002**
**Smoke habits (%, n)**					0.71
Current smoker	43.6 (24)	44.7 (34)	34.9 (22)	28.6 (8)	
Former smoker	47.3 (26)	48.7 (37)	55.6 (35)	64.3 (18)	
No smoker	9.1 (5)	6.6 (5)	8.1 (18)	7.1 (2)	
**Atopy (%, n)**	20 (11)	18.4 (14)	15.9 (10)	21.4 (6)	0.91
**Familiarity (%, n)**	23.6 (13)	11.8 (9)	33.3 (21)	10.7 (3)	**0.008**
**Work exposure (%, n)**	32.7 (18)	38.2 (29)	39.7 (25)	3.6 (1)	**0.004**
**Comorbidities (%, n)**					
Arterial hypertension	70.9 (39)	64.5 (49)	71.4 (45)	50 (14)	0.19
Ischemic cardiomyopathy	12.7 (7)	31.6 (24)	9.5 (6)	25 (7)	**0.005**
Atrial fibrillation	9.1 (5)	15.8 (12)	20.6 (13)	17.9 (5)	0.38
Pulmonary hypertension	1.8 (1)	2.6 (2)	1.6 (1)	3.6 (1)	0.93
Asthma	0	5.3 (4)	4.8 (3)	0	0.23
GERD	12.7 (7)	10.5 (8)	11.1 (7)	3.6 (1)	0.62
OSAS	9.1 (5)	21.1 (16)	25.4 (16)	7.1 (2)	**0.04**
Cancer	23.6 (13)	15.8 (12)	19 (12)	21.6 (6)	0.72
CKD	5.5 (3)	11.8 (9)	4.8 (3)	3.6 (1)	0.28
Type2 Diabetes	20 (11)	19.7 (15)	23.8 (15)	28.6 (8)	0.76
Dyslipidemia	41.8 (23)	32.9 (25)	31.7 (20)	25 (7)	0.44
Thyroid disease	18.2 (10)	18.4 (14)	11.1 (7)	14.3 (4)	0.63
Depression	7.3 (4)	7.9 (6)	9.5 (6)	3.6 (1)	0.8
**Total comorbidities (Median, IQR)**	3 [1-4]	3 [1.2-5]	3 [2-4]	2 [1-3.7]	0.55
**Symptoms at first visit (%, n)**					
Dyspnea	81.8 (45)	91.6 (73)	95.2 (60)	82.1 (23)	**0.009**

Continued on next page

Table S1: Clinical and functional features of the overall cohort according to GOLD classes. (Continued)

	GOLD A	GOLD B	GOLD E	GOLD D	P value
Cough	52.7 (29)	52.6 (40)	63.5 (40)	50 (14)	0.49
Phlegm	50.9 (28)	51.3 (39)	57.1 (36)	46.4 (13)	0.79
Wheezing	1.8 (1)	0	3.2 (2)	0	0.38
Weight loss	0	3.9 (3)	0	0	0.12
**CAT (Median, IQR)**	6 [4-8]	16 [13-23]	15 [12-21]	11 [8.2-15.7]	<**0.0001**
**mMRC (Median, IQR)**	1 [1-1]	3 [2-3]	2 [2-3]	1 [1-2]	<**0.0001**
**Exacerbations (%, n)**	16.4 (9)	26.3 (20)	96.8 (61)	17.9 (5)	<**0.0001**
**Exacerbations with hospital admission (%, n)**	0	0	28.6 (18)	0	<**0.0001**
**Lung function**					
FEV1 (%, Mean, SD)	75.5 ± 17.9	61.3 ± 18.1	58.1 ± 19.9	67.9 ± 18	<**0.0001**
FVC (%, Mean, SD)	94 ± 17.9	77.9 ± 20.9	78.3 ± 17.5	88.2 ± 18	<**0.0001**
FEV1/FVC (%, Mean, SD)	60.2 ± 6.9	58.4 ± 12.7	53.4 ± 12.7	55.3 ± 12	<**0.0001**
RV/TLC (%, Median, IQR)	122 [112-133]	135 [116-156.8]	134 [125-156]	127 [110-145.5]	**0.0001**
**Treatments (%,n)**					
LAMA	34.5 (19)	7.9 (6)	7.9 (5)	14.3 (4)	**<0.0001**
LAMA + LABA	32.7 (18)	28.9(22)	12.7 (8)	31.1 (9)	**0.04**
LAMA + LABA + ICS	18.2 (10)	44.7 (34)	73 (46)	42.9 (12)	**<0.0001**
OCS	1.8 (1)	2.6 (2)	6.3 (4)	3.6 (1)	0.56
LTOT	1.8 (1)	34.2 (26)	33.3 (21)	10.7 (3)	**<0.0001**
N-Acetylcysteine	5.5 (3)	9.2 (7)	22.2 (14)	3.6 (1)	0.01

Table S2. Radiological features of the overall cohort according to GOLD classes.

	GOLD A	GOLD B	GOLD E	GOLD D	P value
**HRCT assessed (%, n)**	63.6 (35)	64 (48)	85.7 (54)	78.6 (22)	**0.01**
**HRCT findings (%, n)**					
Emphysema	36.4 (20)	40 (30)	55.6 (35)	39.3 (11)	0.14
GGO	3.6 (2)	8 (6)	14.3 (9)	10.7 (3)	0.24
Consolidations	3.6 (2)	4 (3)	12.7 (8)	21.4 (6)	**0.01**
Septal thickening	14.5 (8)	13.3 (10)	14.3 (9)	10.7 (3)	0.97
Lung scars	21.8 (12)	18.7 (14)	28.6 (18)	7.1 (2)	0.13
Bronchiectasis	12.7 (7)	13.3 (10)	23.8 (15)	14.3 (4)	0.3
Mosaic attenuation	1.8 (1)	4 (3)	3.2 (2)	3.6 (1)	0.92
Multiple nodules	10.9 (6)	10.7 (8)	20.6 (13)	32.1 (9)	**0.03**
Solitary nodules	14.5 (8)	16 (12)	17.5 (11)	14.3 (4)	0.97
Pleural effusion	0	1.3 (1)	3.2 (2)	0	0.44

Table S3. Comparison of arterial blood gas analysis and 6 minute walking test according to GOLD classes.

	GOLD A	GOLD B	GOLD E	GOLD D	P value
**Arterial blood gas analysis**					
pH (median, IQR)	7.43 [7.42-7.44]	7.43 [7.41-7.45]	7.43 [7.42-7.45]	7.43 [7.41-7.44]	0.84
PaO_2 (mmHg, mean, SD)	86 $\pm$ 9.3	78.3 $\pm$ 11.9	74.8 $\pm$ 12.4	80.4 $\pm$ 11.8	$<$**0.0001**
$PaCO_2$ (mmHg, median, IQR)	39 [36-41.6]	40 [37-43.2]	41.5 [38-45]	39 [37.5-43]	0.12
HCO_3^- (mEq/L, median, IQR)	25.7 [24.2-27.9]	26.8 [24.5-28.1]	27.4 [25.5-29.2]	26 [25-27.5]	0.08
**6MWT**					
Pre-test Borg scale (median, IQR)	0 [0-0]	0.25 [0-1.25]	0 [0-1]	0 [0-1.75]	0.07
Post-test Borg scale (median, IQR)	2 [1-3]	4 [2-7]	4 [2-5]	3 [2-4.75]	**0.005**
Pre-test SpO_2 (%, median, IQR)	97 [95-97]	95.5 [94-97]	97 [95-97]	97 [95.2-98]	**0.04**
Post-test SpO_2 (%, median, IQR)	93 [95-92]	92 [89-95]	92.5 [89.2-95]	94 [91-95]	0.3
Pre-test HR (bpm, mean, SD)	77.5 $\pm$ 12.9	81.5 $\pm$ 13	80.1 $\pm$ 16.4	75.4 $\pm$ 19.1	0.51
Post-test HR (bpm, mean, SD)	93.8 $\pm$ 18.1	96.4 $\pm$ 20.4	95 $\pm$ 24.3	93.3 $\pm$ 17.8	0.95
6MWD (m, median, IQR)	460 [391.5-501]	310 [212-440]	350 [166-450]	445 [352.5-485]	$<$**0.0001**
