# Supplementary figures and images for: Real-Life Characteristics of Patients with COPD and Discordant CAT-mMRC Questionnaires
Source: J Clin Med. 2025 Dec 11;14(24):8771. doi: 10.3390/jcm14248771 (PMC12733573; doi:10.3390/jcm14248771)

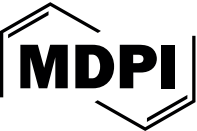

Supplement: Supplementary file 1 [file jcm-14-08771-s001.zip › Definitions/logo-mdpi-eps-converted-to.pdf]

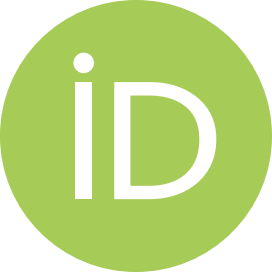

Supplement: Supplementary file 1 [file jcm-14-08771-s001.zip › Definitions/logo-orcid.pdf]
